# Investigation of optical, dielectric, and conduction mechanism in lead-free perovskite CsMnBr_3_†

**DOI:** 10.1039/d4ra01151a

**Published:** 2024-03-27

**Authors:** Moufida Krimi, Fadhel Hajlaoui, Mohammed S. M. Abdelbaky, Santiago Garcia-Granda, Abdallah Ben Rhaiem

**Affiliations:** a Laboratory LaSCOM, Faculty of Sciences of Sfax, University of Sfax BP1171 3000 Sfax Tunisia abdallahrhaiem@yahoo.fr; b Laboratoire Physico-chimie de l’Etat Solide, D'epartement de Chimie, Facult'e des Sciences de Sfax, Universit'e de Sfax B.P. 1171 3000 Sfax Tunisia; c Department of Physical Chemistry, Faculty of Chemical Sciences, University of Salamanca E-37008 Salamanca Spain; d Department of Physical and Analytical Chemistry, Faculty of Chemistry, University of Oviedo-CINN(CSIC) 33006 Oviedo Spain

## Abstract

Metallic perovskites have advantageous optical and electrical properties, making them a valuable class of semiconductors for the manufacturing of solar cells. CsMnBr_3_ is notable among them due to its important optical characteristics. The electrical and dielectric characteristics as a semiconductor are examined in this study. Direct transitions with a 3.29 eV bandgap and an Urbach energy of 0.96 eV are revealed by the results. Through AC conductivity, it demonstrated semiconductor characteristics at 443 K. The dielectric loss varied with frequency and peaked at high frequencies. Furthermore, as temperature rose, a relaxation peak in the electrical modulus was seen to migrate to higher frequencies. Ac conductivity is described by the double power law expression. The conduction in our compound is governed by small polaron tunneling. Based on the optical results reported in the bibliography for this sample, we realize the importance of examining the electrical characteristics to comprehend the semiconductor behavior of CsMnBr_3_.

## Abstract 2

Metallic perovskites have advantageous optical and electrical properties, making them a valuable class of semiconductors for the manufacturing of solar cells.

## Graphical abstract

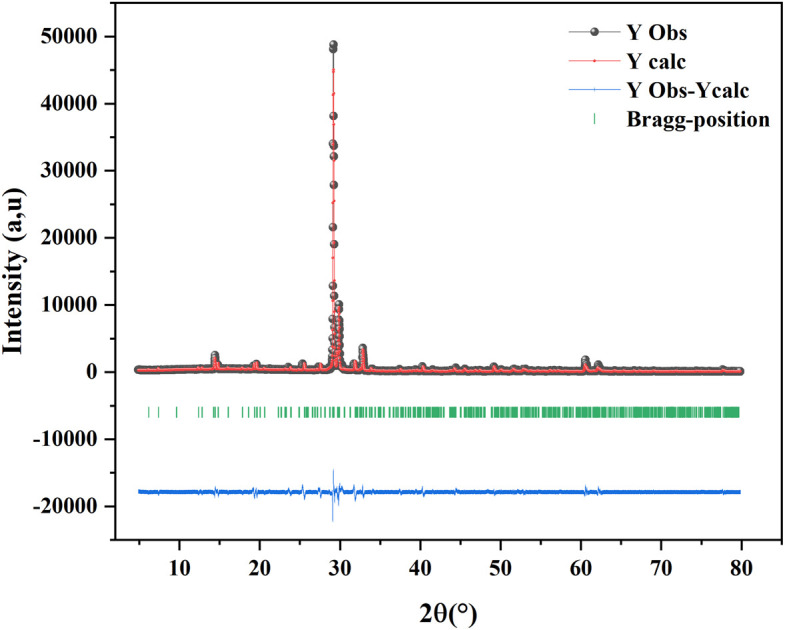

## 1. Introduction

Perovskite-based solar cells, especially those containing lead-free metal halides, have significantly improved their energy efficiency, jumping from 3.8% in 2009 to 25.7% in 2022.^1^ However, the use of these materials is strictly regulated due to their harmful effects on human and animal health, as well as the environment, and their long-term instability. To tackle these challenges, the design of stable lead-free perovskites is a promising approach. These alternative materials have shown great potential for various green energy applications, including photovoltaic solar cells. Many researchers have focused on the fabrication and study of the optical/optoelectronic properties of halogenated metal perovskites, as they play a crucial role in the efficiency of electrical and optoelectronic devices by facilitating charge transport. Many researchers have focused on studying the optical/optoelectronic properties of metal halide perovskite materials, which are crucial for the efficiency of electrical and optoelectronic devices due to their role in charge transport. The optical properties of metal halide perovskites have been found to be highly tunable, making them attractive for a wide range of applications in photovoltaics, light-emitting diodes, and photodetectors.^2–4^ Researchers have been investigating ways to enhance the absorption and emission properties of these materials, as well as improve their stability and performance under different environmental conditions. Additionally, the understanding of charge transport mechanisms in metal halide perovskites is crucial for the development of efficient electronic devices with high conductivity and low recombination rates. However, there is still a lack of complete understanding regarding the electrical transport mechanism and dielectric behavior. Mohammed *et al.*^5,6^ conducted a study in this area, uncovering that metal halide perovskite demonstrates a significant dielectric constant with minimal dielectric loss. The CsSnCl_3_ compound exhibits a dielectric constant of 10^4^, surpassing that of MCdCl_3_ (M = CH_3_NH_3_, (CH_3_)_2_NH_2_), which is at 10^3^. This variation in dielectric properties is also reflected in the difference in electrical conductivity (10^−2^ Ω cm^−1^ for CsSnCl_3_ and 10^−4^ Ω cm^−1^for MCdCl_3_).^5,6^ These materials have a significant energy storage capacity per unit volume due to their high dielectric constant. Additionally, their low dielectric loss (<0.1) allows them to store energy for long periods. These characteristics make them potentially interesting materials for energy recovery devices, like high-performance capacitors or energy storage systems for electronic applications. Further research in this area could provide valuable insights into the underlying mechanisms governing the unique dielectric and electric behavior of metal halide perovskites, ultimately leading to the development of advanced electronic devices with enhanced performance characteristics. CsMnBr_3_ is a well-studied compound in the realm of optoelectronic devices. This perovskite specimen displays notably short radiative lifetimes (in the picosecond range) and a high photoluminescence quantum yield (PLQY) of 54%, emitting a red photoluminescence response.^7^ In addition, CsMnBr_3_ exhibits stronger electronic and longitudinal optical phonon coupling strength than that of NCs at low temperatures. SC has a strong saturable absorption property, with a modulation depth of approximately 27%. Interestingly, the SC also exhibits a large two-photon absorption coefficient of about 0.035 cm GW^−1^ at 800 nm and excellent optical limiting behavior. The experimental results indicate that this material is a class of excellent eco-friendly optoelectronic materials.^7^ The lack of understanding surrounding the electric and dielectric properties of CsMnBr_3_ is the central focus of our investigation that's why our research is dedicated to exploring the electrical and dielectric traits of CsMnBr_3_.

To achieve this goal, a series of experiments were carried out to analyze the conductivity and dielectric features of CsMnBr_3_ under various temperature and frequency conditions. Our results unveil intriguing patterns in the material's behavior, offering insights into its potential applications.

## 2. Experimental

### 2.1. Synthetic processes

The CsMnBr_3_ single crystal was obtained using a slow evaporation method. Briefly, 0.5 mmol (0.106 g) of CsBr and 1 mmol (0.224 g) of MnBr_2_ were added to the mixed solution of water and hydrobromic acid. The obtained solution is then stirred at a temperature of 120 °C for one hour then the mixture is allowed to evaporate at room temperature. After being stored for 5 days, a polycrystalline material with white crystal of mm size was formed as shown in Fig. (S1).†

### 2.2. Equipment

The purity of the compounds is checked using X-ray powder diffraction at the ambient temperature with CuKα radiation (*λ* = 1.5406 A, 10° ≤ 2*θ* ≤ 80°). Furthermore, UV-visible spectroscopy was utilized to analyze the optical characteristics of the material under investigation. The analysis was conducted on a 1 mm thick pellet employing the “UV-3101PC” within the wavelength range of 200–800 nm. The electrical impedance of a silver-covered pellet, 1 mm thick with an 8 mm surface area, was measured across temperatures ranging from 363 K to 473 K and frequencies from 0.1 to 10 million Hertz using a “Solartron1260” device. To enhance accuracy, the pellet was coated with a layer of silver. The measurement involved utilizing two thin copper wires with silver paint for electrical contacts on the pellet's surface. This setup allowed for impedance measurements within a specific temperature and frequency range, ensuring precise data collection.

## 3. Result and discussion

### 3.1. X-Ray diffraction

**Fig. 1:**
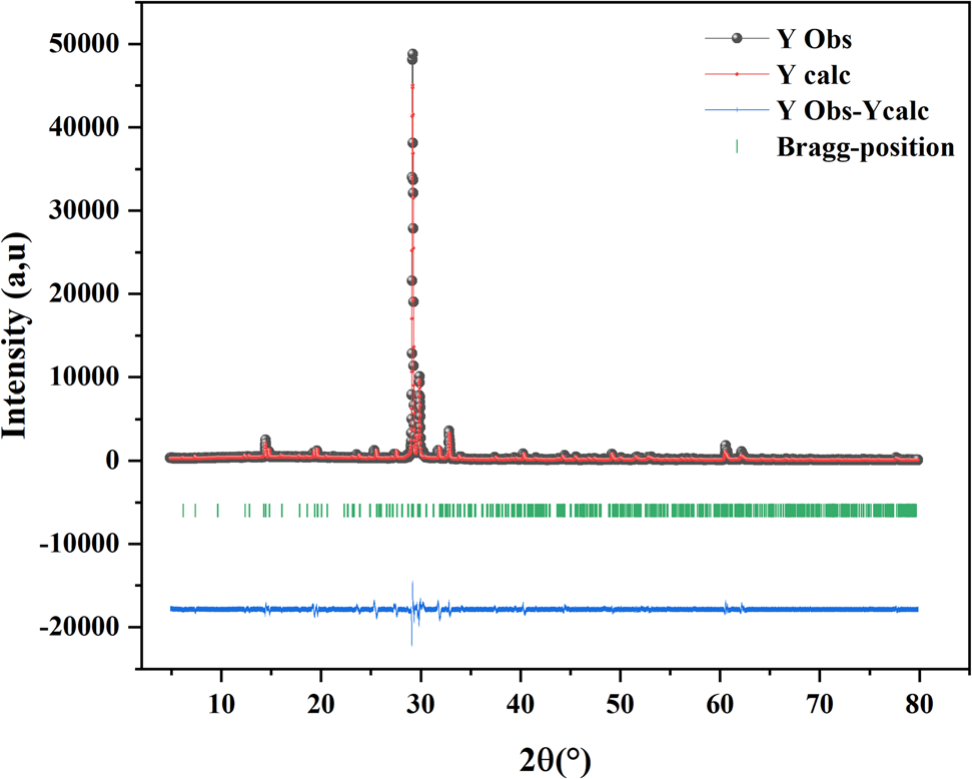
The X-ray pattern of CsMnBr_3_ powdered sample.

The final result for X-ray Rietveld refinement of the powdered sample of our compound is presented in Fig. 1. As shown the observed data matches with the expected diffraction patterns and unit cell parametersobtained from DICVOL indexing, Le Bail fitting, and the Rietveld refinement using FULLPROF in WinPLOTR, emphasizing the strong evidence supporting the presence of the title compound as the sole phase within the analyzed batch.

The obtained phase was fully indexed in the *P*6_3_/*mmc* space group of the hexagonal crystal structure (JCPDS No. 26-0387). The unit cell parameters as well as the fit criteria are reported in the following table:

The crystal structure of CsMnBr_3_consist of linear chains of distorted face-sharing [MnBr_6_]octahedron with a *D*_3d_ symmetry parallel to the *c*-axis that are bridged by Cs ions, this later is bound to twelve equivalent Br^−^ atoms to generate CsBr_12_ cuboctahedra. These cuboctahedra share corners with six others equivalent CsBr_12_ cuboctahedra, faces with eight others equivalent CsBr_12_ cuboctahedra, and faces with six others equivalent MnBr_6_ octahedra. In CsBr_12_ there are six long Cs–Br bonds of length 3.98 (1) Å and six short bonds of length 3.83 (2) Å. In this material the Mn–Br bonds are shorter than those of Cs–Br which are of the order of 2.69 (3) Å.^8,9^

**Fig. 2:**
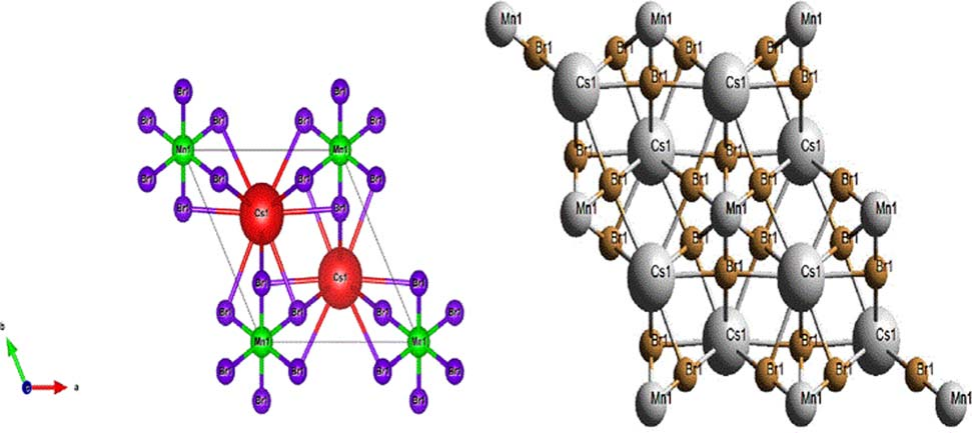
Schematic representation of the CsMnBr_3_ structure.

As seen in Fig. 2, the compound forms chains of face-sharing octahedral composed of manganese surrounded by six bromine atoms [MnBr_6_], those atoms are arranged in a manner creating extended chains along the crystallographic *c*-axis.The arrangement of cesium ions between the [MnBr_6_] chains ensures the overall charge neutrality of the crystal lattice. This ionic arrangement stabilizes the structure, preventing excess positive or negative charges within the unit cell. The chains of [MnBr_6_] octahedra, along with the cesium ions, are interconnected in three dimensionsresulting in a networked structure.

### 3.2. Optical properties

UV-visible spectroscopy is a technique used to analyze how molecules interact with light in the UV and visible regions of the electromagnetic spectrum. It provides information about the electronic transitions and absorption of light by molecules, offering insights into their chemical structure, concentration, and properties. This study is employed to estimate the band gap of materials, especially semiconductors or insulators, by analyzing their absorption spectra. The band gap represents the energy difference between the valence band (highest energy level filled with electrons) and the conduction band (lowest energy level with no electrons) in a material's electronic structure. Also, this measurement serves to the calculation of the absorption coefficient (*α*) which allows us to study the optically induced transition and to better understand the band structure of ourmaterial.

**Fig. 3:**
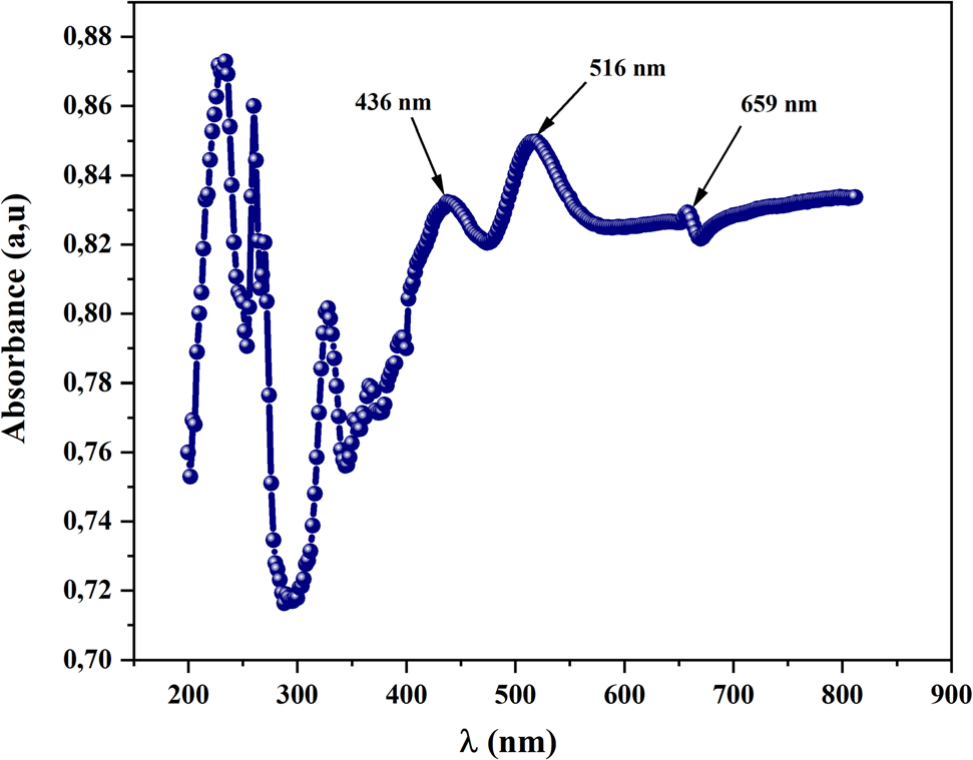
Absorbance spectrum of CsMnBr_3_ compound.

To investigate the optical properties of CsMnBr_3_ compound, we recorded the absorbance spectra in the UV-visible region 200–900 nm at room temperature. Fig. 3 illustrates the absorbance spectrum *versus* wavelength of the obtained device. In ultra-violet range, (*λ* < 400 nm), we notice the presence of an absorption band at *λ* = 326 nm was attributed to the ligand–metal transition (LMCT) of the perovskite sample.^10^ In fact, CsMnBr_3_ consists of cesium cations (Cs^+^) and manganese(iii) ions (Mn^3+^) that are coordinated with bromine ions (Br^−^) in a crystal structure. The manganese ion is the transition metal in this compound, and the LMCT transitions would typically involve the interactions between the d orbitals of manganese and the p orbitals of bromine.^11^ Furthermore, in the wavelength higher than 400 nm, three main strong absorption peaks at 436 nm, 516 nm and 659 nm are depicted. Those peaks can be attributed to the electronic transition of Mn (3d).^12^ Those properties allow our compound to be used as a photo-catalysis material and photovoltaic solar cell.^13^

**Fig. 4:**
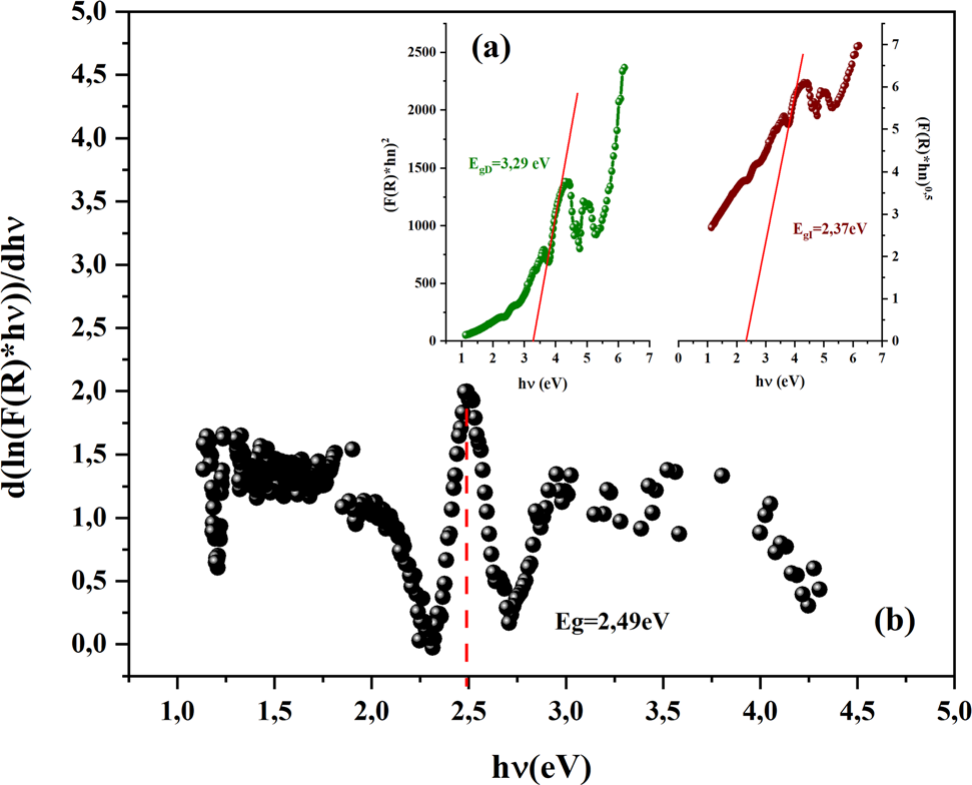
(a and b): a dependence of (*F*(*R*) × *hν*^2^) and (*F*(*R*) × *hν*^1/2^) on the energy (*hν*).

Reflectance spectrum can be used to calculate the optical band gap for such compound. This key parameter is essential for designing electronic devices. For instance, in solar cells or photodetectors, the band gap influences the absorption of light and the efficiency of converting photons into electrical energy. Accurate knowledge of the band gap helps optimize the design and performance of such devices. Calculate the optical band gap for CsMnBr_3_ compound is essential for applications in light-emitting diodes (LEDs), lasers, sensors, and other optical devices. In this work the calculation of the band gap (*E*_g_) is done based on the Kubelka–Munk equation:^14,15^
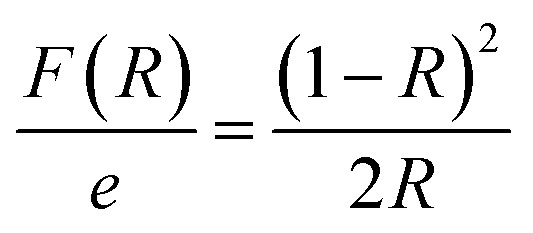
where *R* is the reflectance and *ε*′ is the thickness of the compound (*e* = 1 mm), the term (*F*(*R*)/*e*) is proportional to the absorption coefficient (*α*). In order to calculate the *E*_g_, the Kubelka–Munk modified equation was used by multiplying *F*(*R*) by (*hν*) and using the coefficient (*n*) associated with an electronic transition as mentioned below:
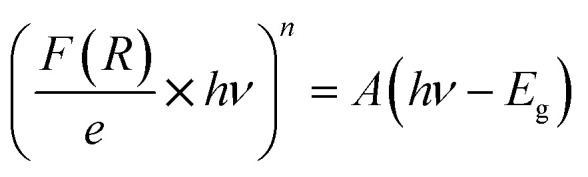
With *n* is a number that identifies the type of the transition. Where *n* = 1/2 for an indirect allowed transition, *n* = 2 for an allowed direct transition, *n* = 2/3 for direct forbidden transition and finally *n* = 1/3 for indirect forbidden transition.^16^Fig. 4a shows the dependence of (*F*(*R*) × *hν*^2^) and (*F*(*R*) × *hν*^1/2^) on the energy (*hν*) curves for the CsMnBr_3_ compound.

After extrapolating the linear sections of these graphs to the energy axis, the energy value at (*F*(*R*) × *hv*)^*n*^ = 0 provides the values of the energy gap, *E*_gd_ = 3.29 eV and *E*_gin_ = 2.37 eV. The obtained band gap values were included in the range band gap semiconductors which confirm the importance of our sample for several applications.

Using the logarithmic derivative (LD) we can recalculate the band gap energy there by identifying the transition mode of our studied compound.^17^ LD transformation starts from the modified Kubelka–Munk equation. For the sake of calculating the natural logarithm, let us suppose that all the quantities in the previous expression are unitless then the natural logarithm will be:
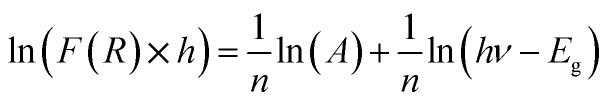
By differentiation of this equation by respect of *hν* we obtain the following formula:
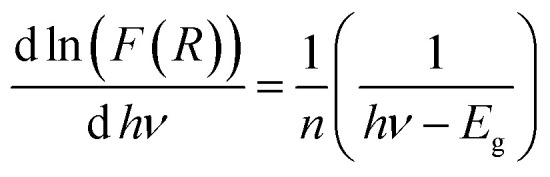


We use the experimental data to calculate the left side of this equation. Drawing the curve of (d ln(*F*(*R*))/d *hν*) *vs.* (*hν*), we can accurately determine the optical band gap value (Fig. 4b). A peak indicates the band gap value of 2.49 eV, which closely aligns with the calculated value of 2.37 eV obtained by extrapolating the Kubelka–Munk expression for the indirect allowed transition.

In a semiconductor or insulating material, the absorption edge typically displays a rapid increase in absorption at photon energies near or above the band gap energy. Beyond this edge, in the tail region of the absorption spectrum, there exists an exponential increase in absorption, often referred to as the Urbach tail. The Urbach tail represents the localized states in the band gap of the material caused by defects, impurities, or structural disorder.^18^ Empirically, Urbach tail Eucan be obtained using the Urbach–Martienssen law presented by the following expression:
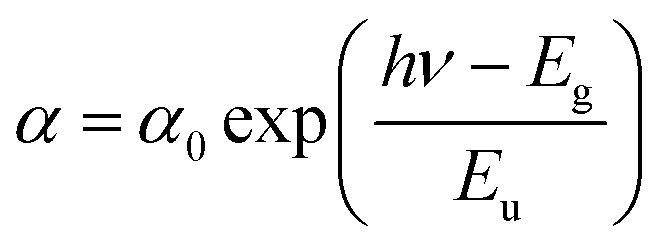
where *α*_0_ is a constant and *E*_u_ is the Urbach energy (eV).

**Fig. 5:**
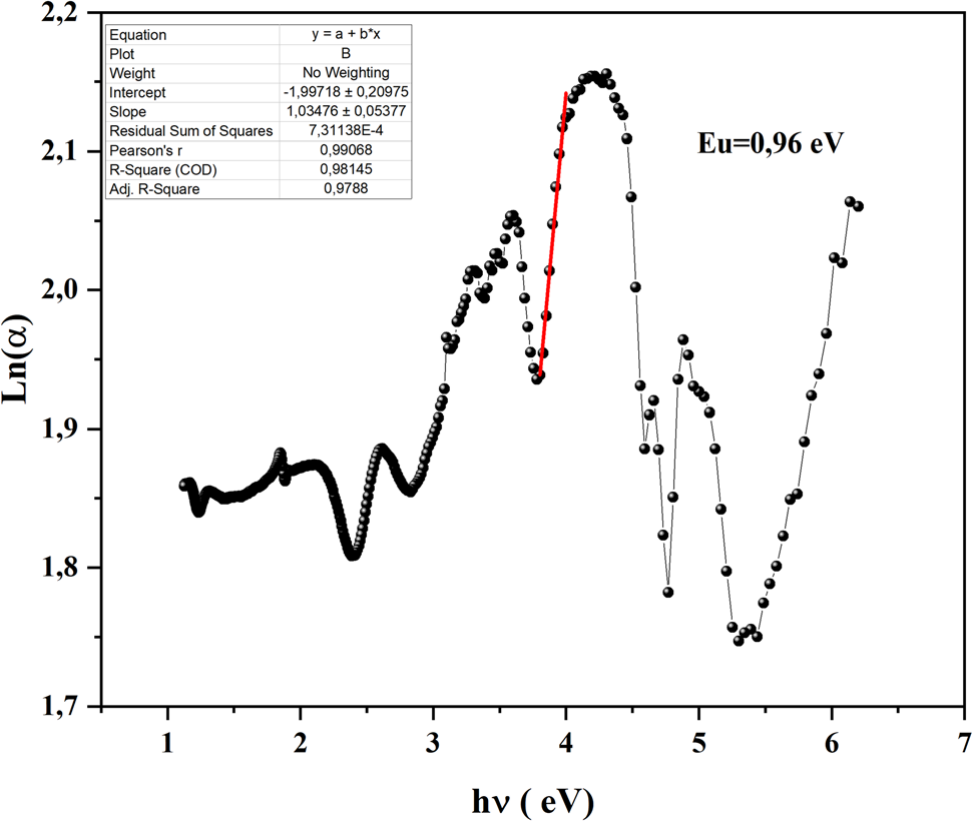
Ln (*α*) as a function of (*hα* (ev)) for CsMnBr_3_ perovskite sample.

We notice that they obtained Urbach-energy (0.96 eV) present only 30% from the band gap energy which indicates a narrower distribution of localized states within the band gap (Fig. 5). This implies lower disorder, fewer defects and reduced structural imperfections within CsMnBr_3_ material. The Urbach energy can be more exploited using the following expression:
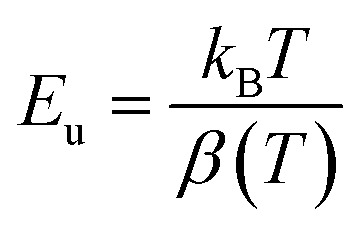
where *k*_B_ is the Boltzmann's constant, *T* = 300 K is the absolute temperature and *β*(*T*) describes how steeply the absorption edge broadens in a material due to the interaction between electrons and phonons within the band gap,^19^ it was found *β*(*T*) = 0.027. This parameter is related to the strength of the electron–phonon interaction (*E*_e–ph_) by the expression:^20^
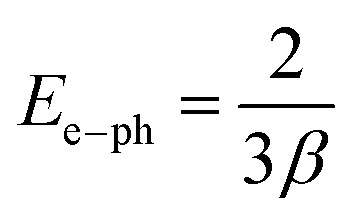


The estimated values of electron–phonon interaction strength (*E*_e–ph_) are close to 24.7 eV.

### 3.3. Electrical properties

#### 3.3.1 TSM (transition semiconductor–metal) identification

##### 3.3.1.1 AC conductivity

**Fig. 6:**
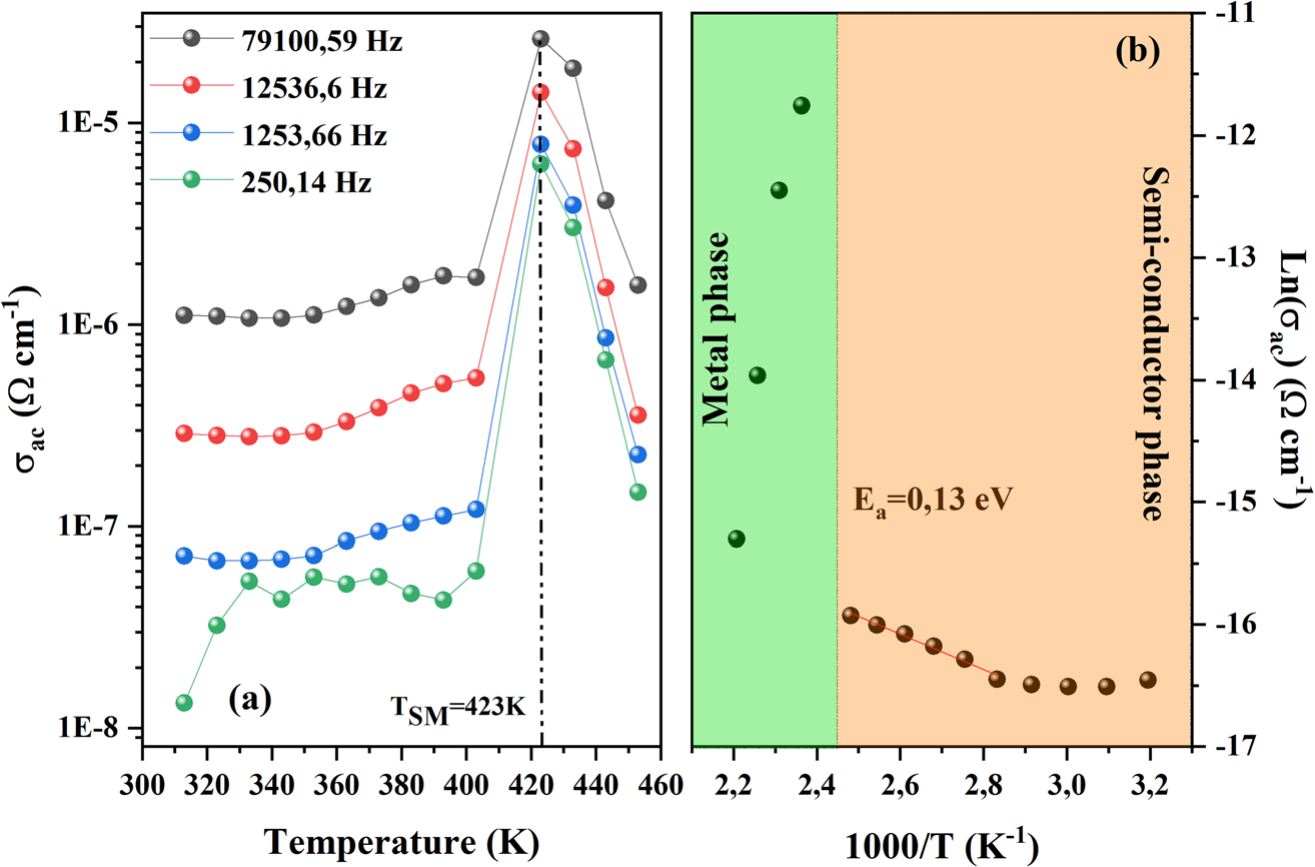
(a) Ac conductivity *vs.* temperature for CsMnBr_3_ perovskite sample. (b) Variation of ln(*σ*_ac_) as a function of 1000/*T*.

AC-conductivity as a function of temperature for the CsMnBr_3_ sample for selected values of frequency is depicted in Fig. 6a. Obviously, the AC conductivity of the compound studied increases then decreases as the temperature varies. Besides, a semiconductor–metal transition^21^ takes place at around TSM = 423 K. Noticeably, the variation below TSM reflects a semiconductor behavior, while that above TSM exhibits metallic behavior. This change in behavior of the conductivity can be due to several factors. Generally, the increase in conductivity with increasing temperature is attributed to the thermally activated charge carrier. In fact, the ionic conductivity of perovskite sample depends on the rate and mobility of charge carriers.^22^ As the temperature increases, traps within the material start to release charge carriers, indeed, with an increase in temperature, the traps (defects and grain boundary) become less effective at holding onto the charge carriers, causing them to be released, increasing the free charge carrier, and contributing to the material's conductivity. However, for such temperature called TSM this emission stop, when all the trapped charges become free leading to saturation in the conductivity process before it starts to decrease with increasing temperatures. This decrease can be explained by the fact that the conductivity depends on the density of available free charge carriers. Some of these carriers might get stuck or trapped at defects in the grain boundaries of the material. Additionally, the movement of these carriers can be affected by vibrations in the lattice, caused by phonons. These vibrations can limit the carrier's ability to move freely, thereby reducing the overall conductivity.

To investigate the effect of the observed transition in the activation energy for the studied compound we fitted the obtained curves of *σ*_ac_ for a fixed value of frequency (12 536.6 Hz) using the Arrhenius expression as depicted in Fig. 6b.
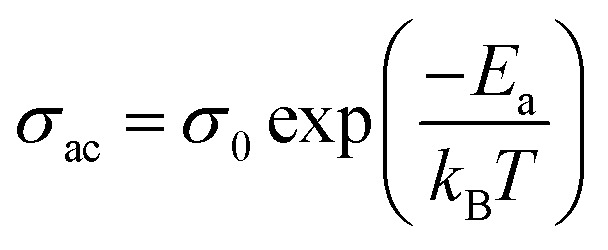
where *k*_B_, *σ*_0_, *T*, and *E*_a_ denote the Boltzmann constant, pre-exponential factor, temperature, and activation energy, respectively.

The observed linear variation in the Arrhenius plot reveals that the observed semiconductor–metal transition is temperature-activated. This transition is filled by a leap in activation energy; the increase in activation energy shows a noticeable shift in the material's conducting qualities as temperature increases. The lower activation energy of 0.13 eV in the semiconductor state indicates that charge carriers can easily pass over the energy band gap, contributing to the material's semiconductor-like characteristics. However, as the temperature increase the conductivity decreases indicating a change toward metallic behavior.

#### 3.3.2 Electrical properties of the semiconductor phase

##### 3.3.2.1 Impedance spectroscopy (CIS)

Impedance spectroscopy is a powerful tool for studying the electrical behavior of materials under different conditions and frequencies, providing valuable insights into their performance, and enabling the optimization of various technologies and systems. The contribution of electrodes, grain boundaries and grains to charge transport in the material can be differentiated through the complex impedance study. The frequency dependence of the complex impedance is illustrated by the following equation:*Z*(*ω*) = *Z*′(*ω*) − *jZ*′′(*ω*)

**Fig. 7:**
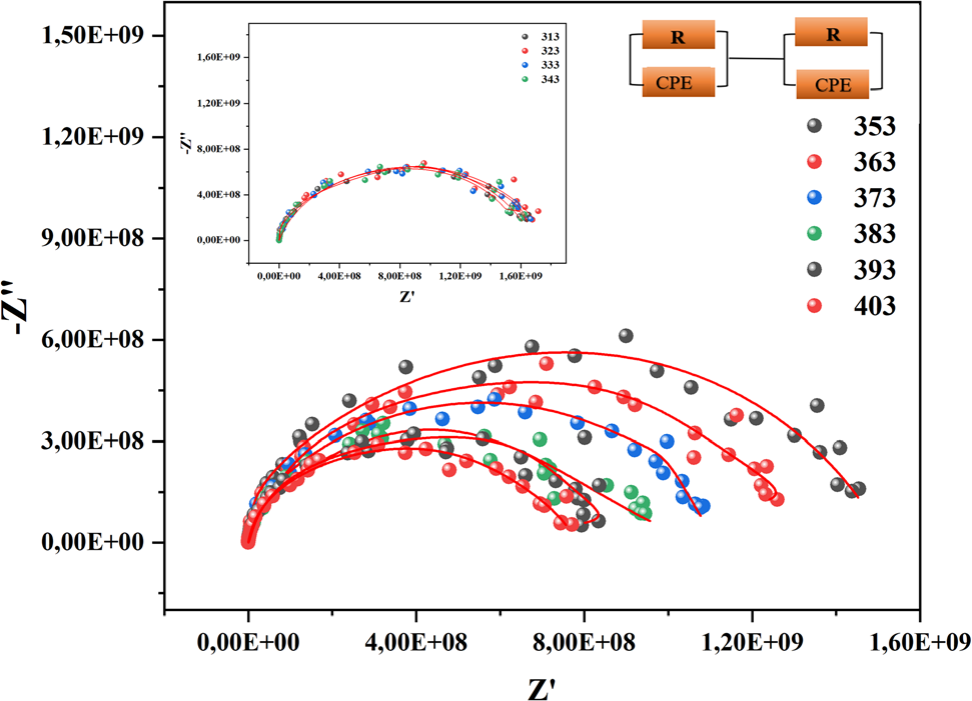
Nyquist diagram of CsMnBr_3_ perovskite sample.

To better differentiate the existing contributions in the material, we draw Nyquist diagrams as shown in Fig. 7.

**Fig. 8:**
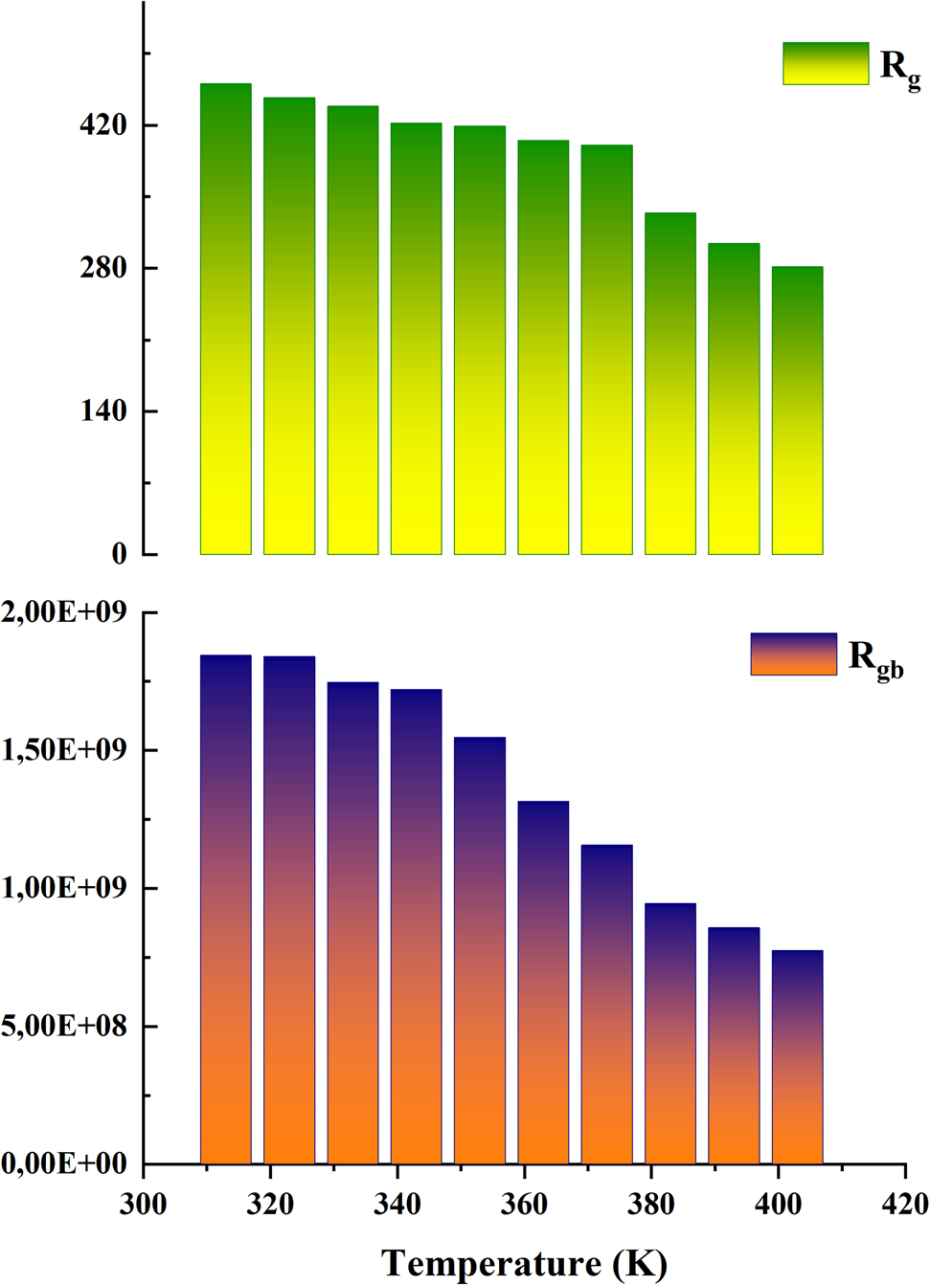
Variation in the strength of the grains and grain boundaries of the CsMnBr_3_ compound.

The analysis of these diagrams clearly shows that the curves showing the variation of (−*Z*′′) as a function of (*Z*′) are centered below the axis of the real of impedance, therefore the conduction of this material does not obey the Debye model, but it follows the Cole–Cole model.^23^ As shown in the inset of Fig. 7 at low temperatures (*T* < 353 K) CsMnBr_3_ compound show only slight variations in impedance, which may be the result of different factors like slow electrode kinetics, limited ion mobility, or reduced system reactivity for that range of temperatures. For temperature greater than 353 K the radius of the semicircles decreases with increasing temperature indicating that the temperature coefficient of resistivity is negative.^24^ This proves that the CsMnBr_3_ compound possess semi-conductive properties and also that the conduction process is thermally activated. The best fit is obtained when employing an equivalent circuit composed by two cellules each one of them modelized by a parallel combination of resistance and fractal capacitance. The semicircle seen at lower frequencies in the impedance spectra illustrate the response of the grain boundaries when the one observed for high values of frequency characterizes the electrical response of the grain. The frequency dependence of *Z*′ and *Z*′′ are shown in Fig. (S2) and (S3).† The strong agreement or good conformity observed between the calculated lines and experimental data suggests that the proposed or suggested equivalent circuit provides a reasonably accurate description of the crystal–electrolyte interface. From the fitted data, specific parameters such as the resistance of both the grain and grain boundary can be extracted. The acquired values of grain and grain boundary resistances as a function of temperature showed in Fig. 8 reveal that the strength of the grains and grain borders diminishes as temperature increases, confirming the previously described negative thermal coefficient of resistance (NTCR) behavior. This behavior is confirmed by the observed decrease in grain and grain boundary resistances with increasing temperature, which can be attributed to many factors such as grain boundary thermal conductivities, grain size effects, thermal resistance and conductance of tilt grain boundaries, and microscale imaging of thermal conductivity suppression.^25^

By comparing the obtained values of resistance for both grain and grain boundary we notice that the resistance associated with the grain is much lower than the resistance associated with the grain boundary, it indicates that the grain is the primary governing species of conductivity. In this case, the decreased resistance in the grain area suggests that charge carriers can pass more freely through the bulk material (grains) rather than being hampered by interfaces or flaws in the grain boundaries.^26^

##### 3.3.2.2 Dielectric permittivity and modulus studies

This compound's dielectric response can be computed using the following formula:*ε*(*ω*) = *ε*′(*ω*) + *jε*′′(*ω*)

The real part, denoted by *ε*′(*ω*), corresponds to the energy stored by the system and *ε*′′ the imaginary component of the equation reflects the energy dissipation of the applied electric field, this have crucial roles in the process of ionic conduction within materials since it is influenced by four main types of polarization: interfacial, dipolar, electronic, and ionic.^27^ The theoretical adjustment of *ε*′′ can be done using several expressions, in our work we use the modified Cole–Cole model us follow:
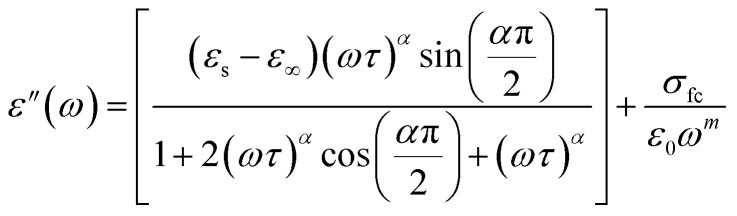
where (*σ*_fc_) represent the free charge carrier conductivity, the high-frequency limit of permittivity is denoted by *ε*_∞_, and the low-frequency limit is designated by *ε*_s_. *τ* indicate the relaxation time. Whereas *α*, the modified Cole–Cole parameter, has a value between 0 and 1 and *m* denotes the frequency exponent. As shown in Fig. (S4)† the dielectric measurement of the CsMnBr_3_ compound is conducted across a broad frequency range from 0.1 to 10^6^ Hz and over a temperature span varying between 353 to 403 K. A first examination of these curves indicates the absence of dipolar relaxation process which can be resulting from the predominance of the electrical conductivity. Also, *ε*′′ shows high value in the low frequency region and revealing a significant value decrease as frequency values progressively increased. This is expected since dipoles, space charges, and ions gradually lose their ability to follow the quickly changing field and stop contributing to the polarization effect as the applied electric field frequency increases.^28^ A good conformity was obtained between the experimental data and the modified cole–cole model yielding the parameters classed in Table (S1).†

We notice that the factor *α* increase with increasing temperature but not reaching the unity (α < 0.724) proving the existence of non-Debye relaxation. Also, the conductivity of free charge *σ*_fc_ increase with increasing temperature whiles the relaxation time decrease. To differentiate between the existed contributions in the material by unmasking the electrode effect we use the electrical modulus, indeed this technique is valuable because it offers insights into the relaxation dynamics and polarization mechanisms of materials. These studies help elucidate how materials respond to an applied electric field across a range of frequencies or temperatures. Being mainly the reciprocal of complex permittivity, the complex modulus expressed by the following expression:
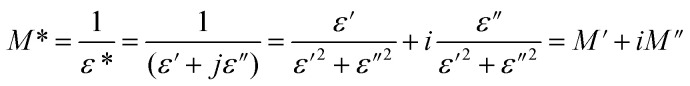
where *M*′, *M*′′, *ε*′, and *ε*′′ refer to the real and imaginary components of the electric modulus (M*) and the dielectric constant (ε*), respectively.

**Fig. 9:**
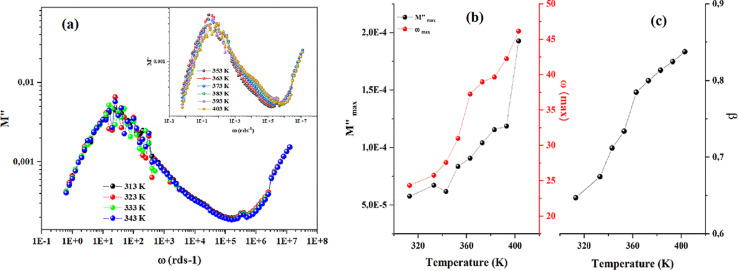
(a) Frequency variation of the imaginary part of complex modulus. (b) Temperature dependency of Bergman's parameter.

As shown in the previous figure, the widths of the asymmetric peaks in the imaginary component of the electric modulus (*M*′′) as a function of frequency are somewhat larger than the Debye peaks. By raising the temperature, the maxima of these peaks move towards the high frequencies. The analysis performed with Bergman's equation validated all these facts:
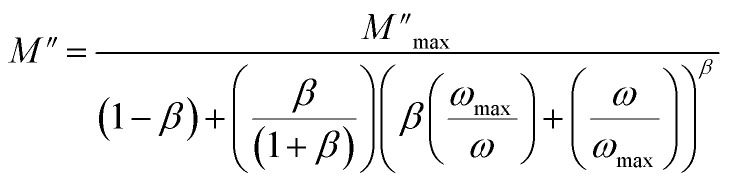
where *β* is the Kohlrausch parameter, which ranges from 0 to 1, and *M*′′_max_ is the maximum of the complex modulus corresponding to *ω*_max_, the variation in temperature of the mentioned parameter is shown in Fig. 9b. Both factor *M*′′_max_ and *ω*_max_ increase with increasing temperature indicating the thermal activated relaxation process. The obtained values of *β* are less than unity indicating the non-Debye relaxation type.

##### 3.3.2.3 AC conductivity

**Fig. 10:**
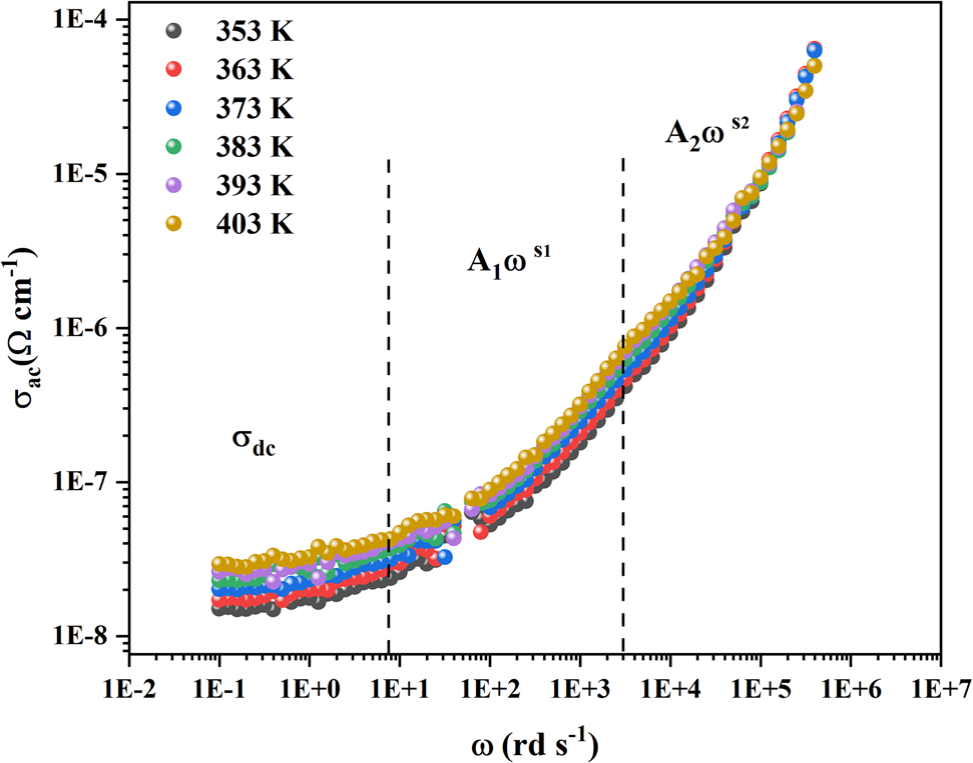
Frequency dependence of ac conductivity of CsMnBr_3_ compound.

The frequency dependence of AC conductivity is depicted in Fig. 10. Three zones are seen by the conductivity curves. The first zone is associated with dc conductivity at low frequencies, whereas the second and third zones correspond to higher frequency values where the conductivity exhibits a dispersion effect, and each is defined by the terms (*Aω*^*s*^).

For such behavior the double power of Jonsher law is used with the following expression:*σ*_AC_ = *σ*_dc_ + *A*_1_*ω*^*s*1^ + *A*_2_*ω*^*s*2^

Were *σ*_dc_ is the pre-exponential factor, *A*_1_ and *A*_2_ are scaling coefficients that describe the amplitude of the frequency-dependent terms 0 < *s*1 < 1, which dominates at low frequencies and corresponds to short motion of the mobile ions. The third term corresponds to the high frequency dispersion region, where 1 *< s*2 *<* 2, that is associated with well localized hopping translational or re-orientational motion.^29^ As presented by Fig. (S6),† a good agreement between the theoretical and the experimental data was obtained which confirm our choice.

**Fig. 11:**
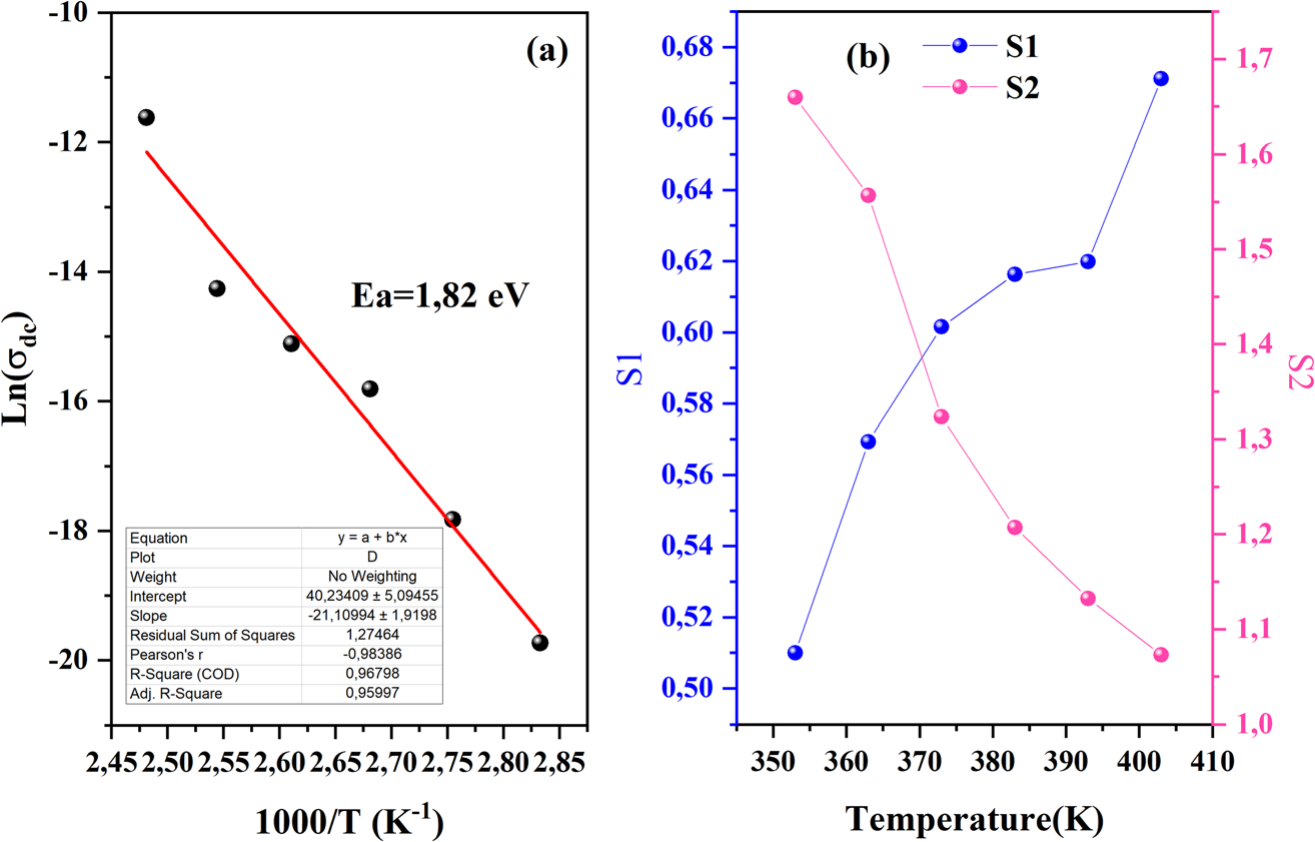
(a) Ln(*σ*_dc_) *vs.* 1000/*T* of CsMnBr_3_ compound. (b) The exponent *s*1 and *s*2 of CsMnBr_3_ compound.

From the previous adjustment, the temperature dependency of *σ*_dc_ was obtained us depicted in Fig. 11a. The single slope of this curve indicates the presence of one single phase in the CsMnBr_3_ compound characterized by an activation energy *E*_a_ = 1.8 eV.

In order to identify the conduction mechanism, we plot the variation of the exponent *s*1 and *s*2 as a function of temperature in Fig. 11b, as presented, the increase in temperature leads to an increase in the values of *s*1 while the value of *s*2 decreases which suggest that the short motion of charge carriers dominates as the temperature increase.^30^ The temperature behavior of *s*1 is typically to that proposed by the non-small polaron tunneling model, for which the conductivity is given by the following expression:^31^
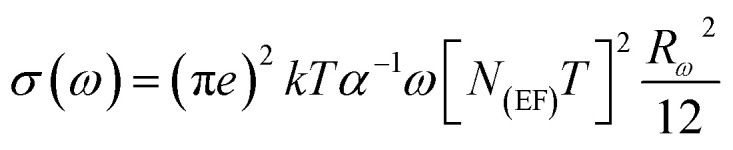
where *α*^−1^ (Å) is the spatial extension of the polaron, *τ*_0_ represents the characteristic relaxations time, whose value is in the order of an atom vibrational period (10^−13^ s), *N*_EF_ is the density of states near the Fermi level and *R*_*ω*_ is the tunneling distance given by the expression:
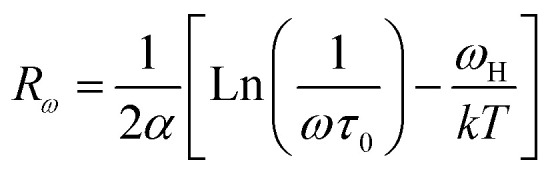
where *ω*_H_ is the energy of polaron hopping. The power exponent is given by the expression:
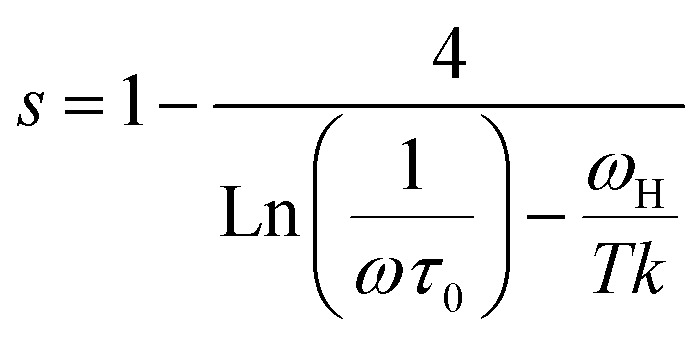


These formulas are employed to adjust the swing of *σ*_ac_ as a function of 1000/*T* for fixed frequency values. As demonstrated in Fig. (S6),† a good agreement was obtained between the empirical model and the experimental data confirming our model choice. The obtained parameters from this adjustment are classified in the Table (S2).†

**Fig. 12:**
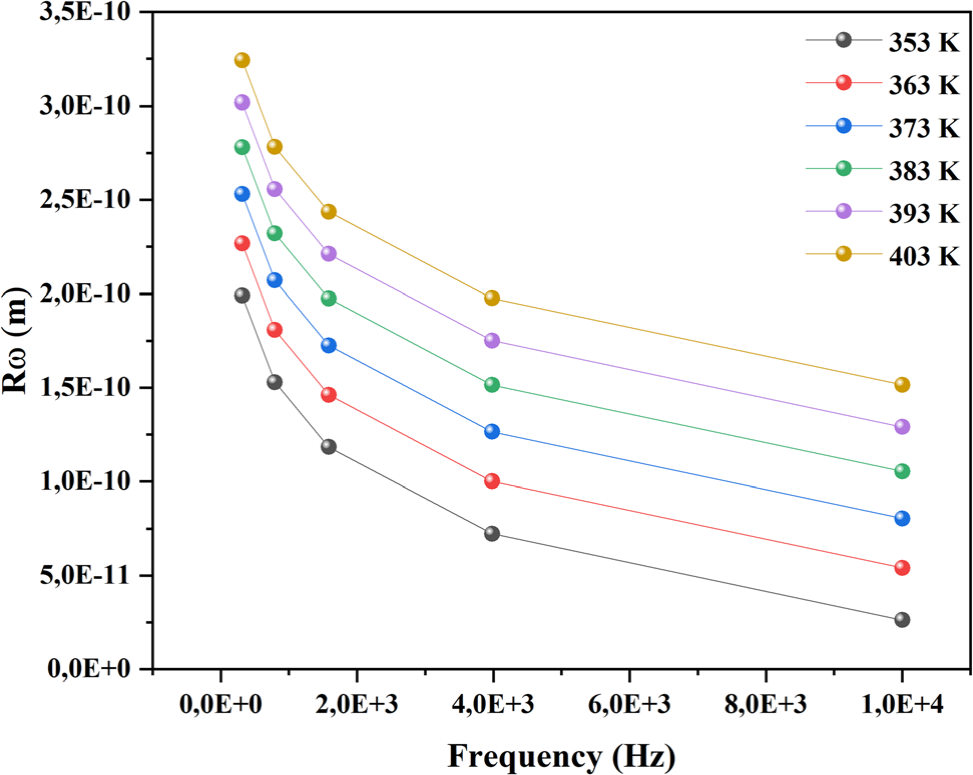
Frequency dependency of *R*_*ω*_ of CsMnBr_3_ compound.

We notice that an increase in frequency leads a decrease in the density of localized states which can be attributed to a change in the electronic state's energy distribution.^32^ Using the obtained value of *ω*_H_ for different frequency values, we can calculate the tunneling distance for the SPT model given by the expression:
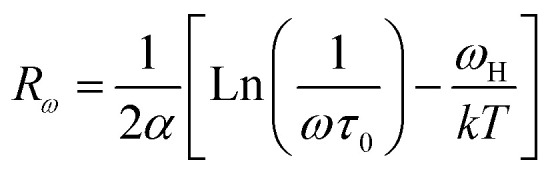


The tunneling distance never goes above 3.5 Å, being less than the interatomic distance, which confirms the dominance of small polaron tunneling. As the frequency increases, the recombination rates (*R*) and the recombination velocity (*v*) decrease, indicating faster lattice oscillations and energy loss. The effective distance that a small polaron may travel before its energy is dissipated is lowered by this energy dissipation, which decreases the tunneling distance (Fig. 12).^33^

## 4. Conclusion

CsMnBr_3_ single crystals with a *P*6_3_/*mmc* space group crystallize in a hexagonal arrangement. According to optical investigation, our compound shows an indirect transition with a bandgap of 2.29 eV, and a disorder indicated by Urbach energy of 0.96 eV, making it a good device for optoelectronic applications. The AC conductivity's temperature dependence exhibits two distinct behaviors: for *T* < 443 K, the conductivity rises as the temperature increases, indicating a semi-conductive behavior; for *T* > 443 K, the conductivity falls as the temperature rises, exhibiting a typical metal behavior. An in-depth examination of the impedance data for *T* < 443 K reveals the grain contribution in addition to the grain boundary. The dielectric study proves that the real part of permittivity is thermally activated which means that increasing the temperature in fact increases the polarized dipoles. This result suggests that the prepared sample is a strong contender for application in FET devices as an active channel or dielectric gate. The frequency dependency of the electrical conductivity shows a double power law type nature. From this study we have demonstrate the NTCR behavior of our compound which leaves our compound compatible with applications based on the detection of infrared rays, also we have extracted the temperature dependency of the exponent S proving that the conduction is governed by the small polaron tunneling mechanism.

## Conflicts of interest

There are no conflicts to declare.

## Supplementary Material

RA-014-D4RA01151A-s001

RA-014-D4RA01151A-s002

RA-014-D4RA01151A-s003
