# Supplementary material for: Investigation of optical, dielectric, and conduction mechanism in lead-free perovskite CsMnBr3
Source: RSC Adv. 2024 Mar 27;14(15):10219–28. doi: 10.1039/d4ra01151a (PMC10968624; doi:10.1039/d4ra01151a)
Supplement: RA-014-D4RA01151A-s001 [file RA-014-D4RA01151A-s001.pdf]

Electronic Supplementary Material (ESI) for RSC Advances.
This journal is © The Royal Society of Chemistry 2024

Investigation of optical, dielectric, and conduction mechanism in lead-free perovskite $CsMnBr_3$.

Moufida Krimi[1], Fadhel Hajlaoui[2], Mohammed S. M. Abdelbaky[3,4], Santiago Garcia-Granda[4] and Abdallah Ben Rhaiem[1*]

[1]Laboratory LaSCOM, Faculty of sciences of Sfax, University of Sfax, BP1171, 3000, Sfax, Tunisia.
[2] Laboratoire Physico-chimie de l'Etat Solide, D´epartement de Chimie, Facult´e des Sciences de Sfax, Universit´e de Sfax, B.P. 1171, 3000 Sfax, Tunisia
[3]Department of Physical Chemistry, Faculty of Chemical Sciences, University of Salamanca, E-37008 Salamanca, Spain
[4]Department of Physical and Analytical Chemistry, Faculty of Chemistry, University of Oviedo-CINN(CSIC), 33006 Oviedo, Spain.
*Author for correspondence (abdallahrhaiem@yahoo.fr)

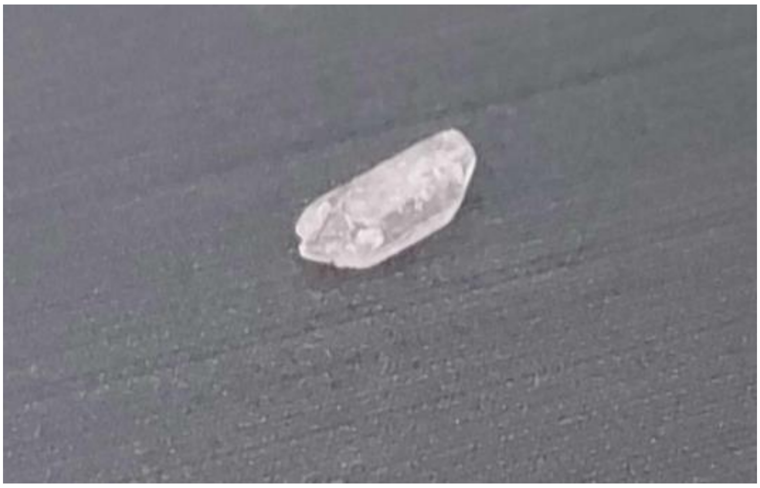

Figure S1: Image of the obtained crystals for $CsMnBr_3$ compound.

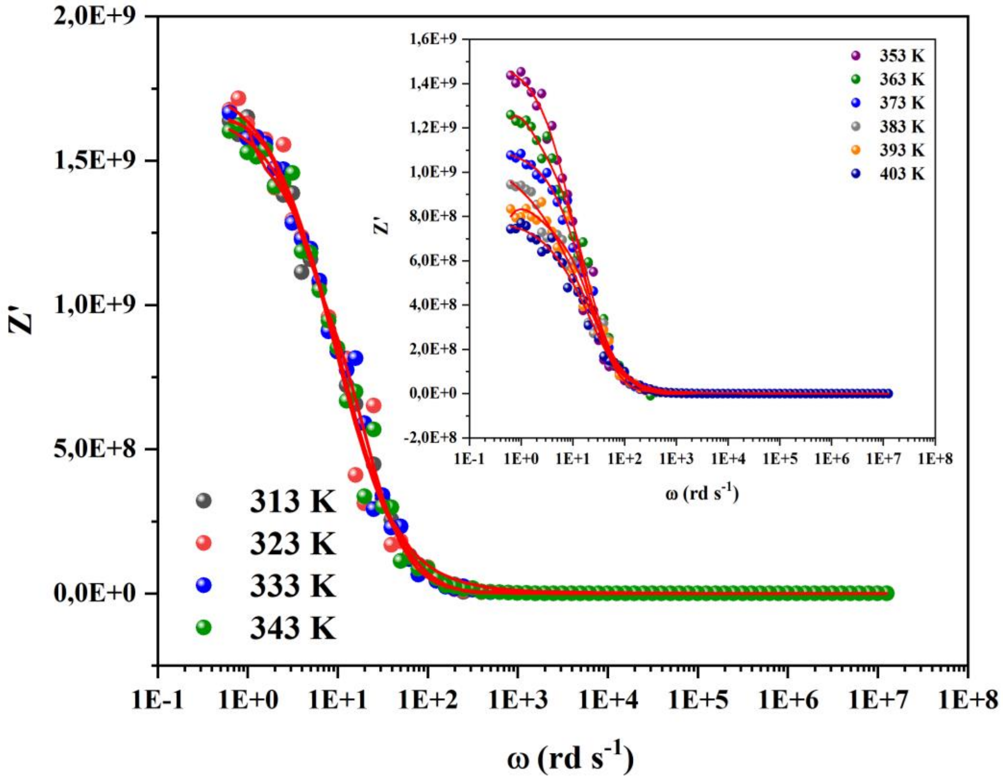

Figure (S2): Frequency dependence of real part of the impedance of $CsMnBr_3$ compound.

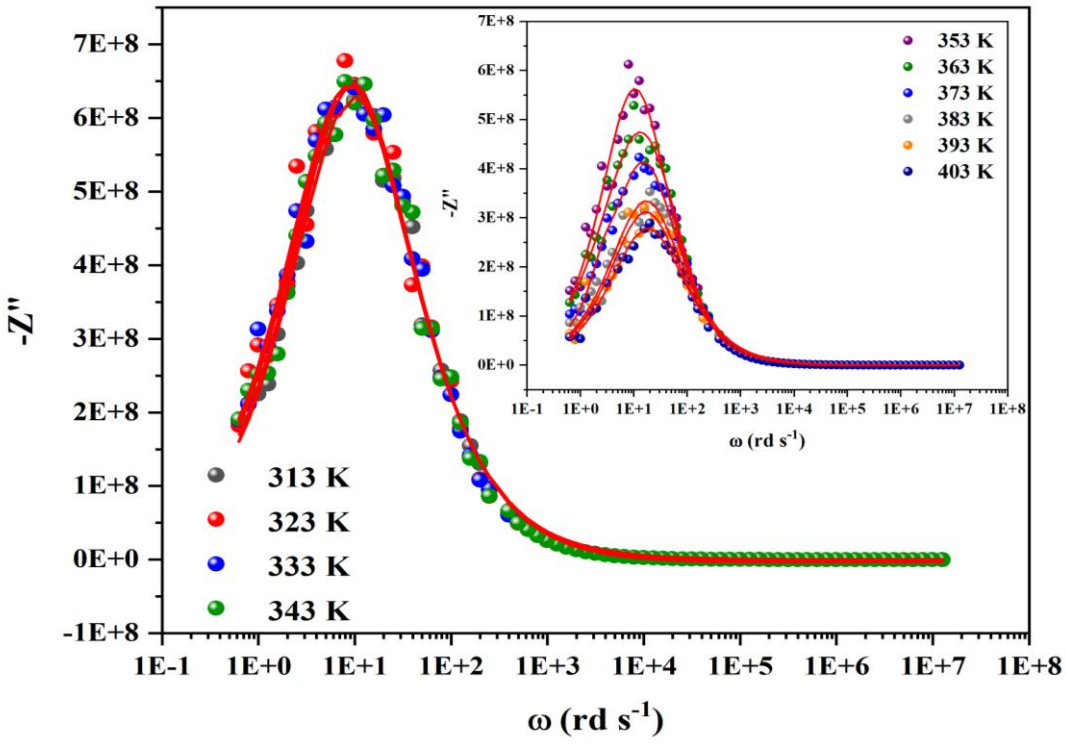

Figure (S3): Frequency dependence of imaginary part of electrical impedance of $CsMnBr_3$ compound.

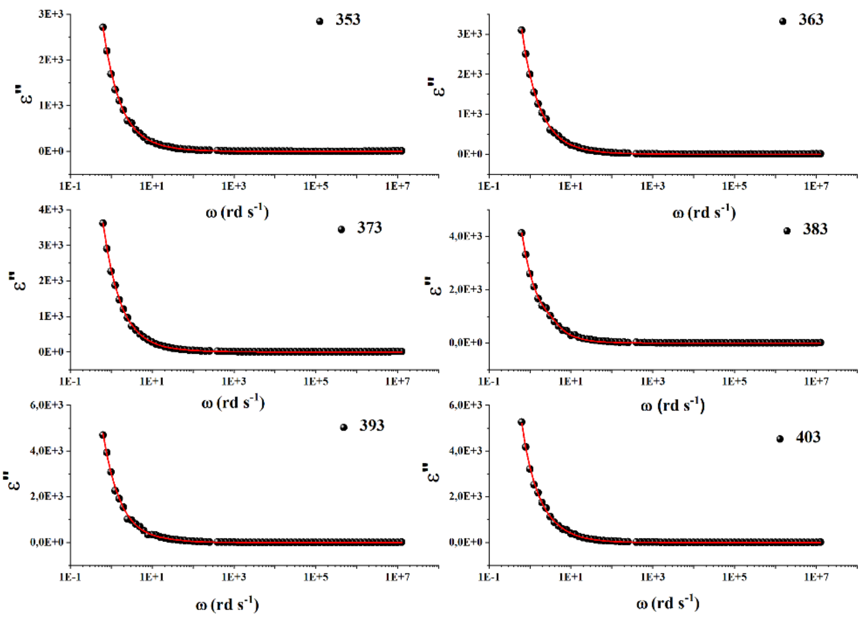

Figure (S4): Frequency-dependent imaginary part of dielectric permittivity at different temperatures.

Temperature (K)	τ	α	M	σ_{fc}
353	**$1.493x10^{-8}$**	**0.325**	**0.425**	**$1.32x10^{-5}$**
363	**$1.324x10^{-8}$**	**0.453**	**0.481**	**$3.635x10^{-5}$**
373	**$1.192x10^{-4}$**	**0.562**	**0.524**	**$4.325x10^{-5}$**
383	**$9.254x10^{-5}$**	**0.584**	**0.632**	**$5.012x10_{-5}$**
393	**$7.326x10^{-8}$**	**0.643**	**0.752**	**$5.832x10^{-5}$**
403	**$5.236x10^{-5}$**	**0.724**	**0.784**	**$6.325x10^{-5}$**

Table S1: The parameters τ , α, m and σ_{fc}evaluated from the fitting of the dielectric loss with the modified Cole - Cole model.

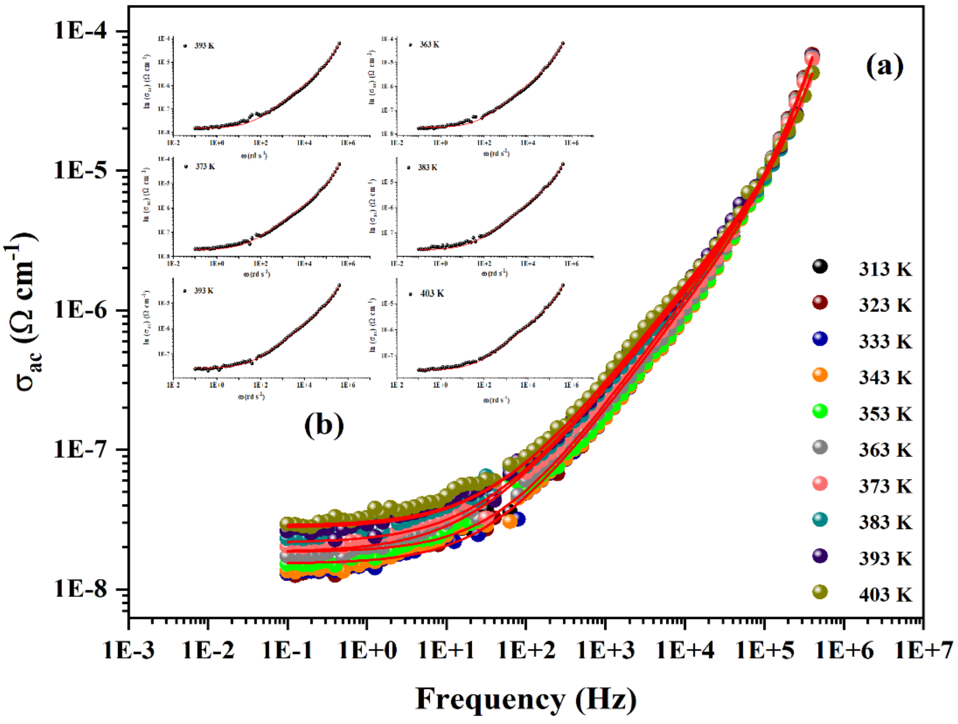

Figure (S5): Fitted curves of AC conductivity of $CsMnBr_3$ compound.

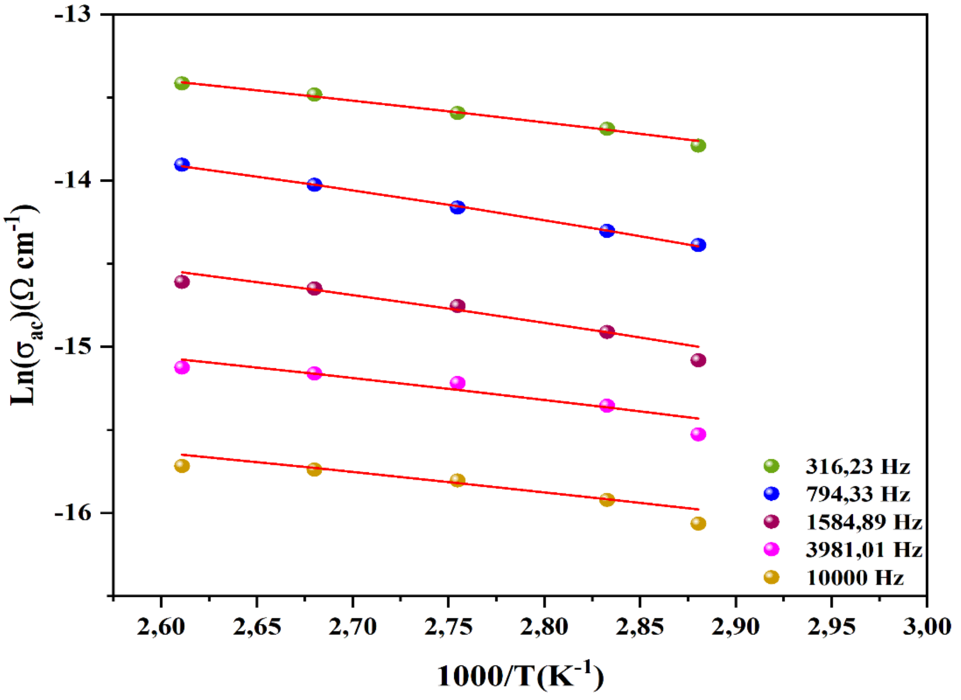

Figure (S6): Temperature dependence of AC conductivity for the CBH model.

**Frequency (Hz)**	**N_T $10^{22}cm^{-3}$)**	**w_H(eV)**
**316.23**	**2.24**	**0.60**
**794.33**	**2.04**	**0.706**
**1584.89**	**1.867**	**0.78**
**3981.01**	**1.77**	**0.74**
**10000**	**1.567**	**0.72**

Table (S2): parameter resulting from NSPT fit.
