# Supplementary material for: Investigation of optical, dielectric, and conduction mechanism in lead-free perovskite CsMnBr3
Source: RSC Adv. 2024 Mar 27;14(15):10219–28. doi: 10.1039/d4ra01151a (PMC10968624; doi:10.1039/d4ra01151a)
Supplement: RA-014-D4RA01151A-s003 [file RA-014-D4RA01151A-s003.pdf]

Electronic Supplementary Material (ESI) for RSC Advances.
This journal is © The Royal Society of Chemistry 2024

Tables

**Crystal system**	**Hexagonal**
**Space group**	**P 63/mmc**
**Formula units (A°)**	***a* = *b* =7.618 (4)**<br>***c* = 6.519 (2)**<br>**$\alpha = \beta = 90^{\circ}$**<br>**$\gamma = 120^{\circ}$**
**Volume ($A^{\circ 3}$)**	**327.637(6)**
**Rp (%)**	**35.5**
**Rwp (%)**	**44.5**
**Rexp (%)**	**29.6**
**χ 2**	**2.23**
**RB (%)**	**5.09**
**RF (%)**	**9.06**

Table 1: Unit cell parameters and fit criteria of $CsMnBr_3$pure polycrystalline material.
